# An optimized two stage U-Net approach for segmentation of pancreas and pancreatic tumor

**DOI:** 10.1016/j.mex.2024.102995

**Published:** 2024-10-04

**Authors:** Himali Ghorpade, Shrikrishna Kolhar, Jayant Jagtap, Jayasree Chakraborty

**Affiliations:** a Symbiosis Institute of Technology, Symbiosis International (Deemed University), Pune, Maharashtra, India; b Marik Institute of Computing, Artificial Intelligence, Robotics and Cybernetics, NIMS University Rajasthan, Jaipur, India; c Department of Surgery, Memorial Sloan Kettering Cancer Center, New York, USA

**Keywords:** Pancreas, Pancreatic tumor, U-Net, Segmentation, Grey Wolf Optimization, Border Collie Optimization, A two-stage hybrid U-Net model with Grey Wolf Border Collie Optimization (GWBCO) method

## Abstract

The segmentation of pancreas and pancreatic tumor remain a persistent challenge for radiologists. Consequently, it is essential to develop automated segmentation methods to address this task. U-Net based models are most often used among various deep learning-based techniques in tumor segmentation. This paper introduces an innovative hybrid two-stage U-Net model for segmenting both the pancreas and pancreatic tumors. The optimization technique, used in this approach, involves a combination of meta-heuristic optimization algorithms namely, Grey Wolf Border Collie Optimization (GWBCO) technique, combining the Grey Wolf Optimization algorithm and the Border Collie Optimization algorithm. Our approach is evaluated using key parameters, such as Dice Similarity Coefficient (DSC), Jaccard Index (JI), sensitivity, specificity and precision to assess its effectiveness and achieves a DSC of 93.33 % for pancreas segmentation. Additionally, the model also achieves high DSC of 91.46 % for pancreatic tumor segmentation. This method helps in improving the diagnostic accuracy and assists medical professionals to provide treatment at an early stage with precise intervention. The method offers

• Two-stage U-Net model addresses both pancreas and tumor segmentation.
• Combination of two metaheuristic optimization algorithms, Grey Wolf and Border Collie for enhanced performance.
• High dice similarity coefficient for pancreas and tumor segmentation.

## Graphical abstract

**Figure:**
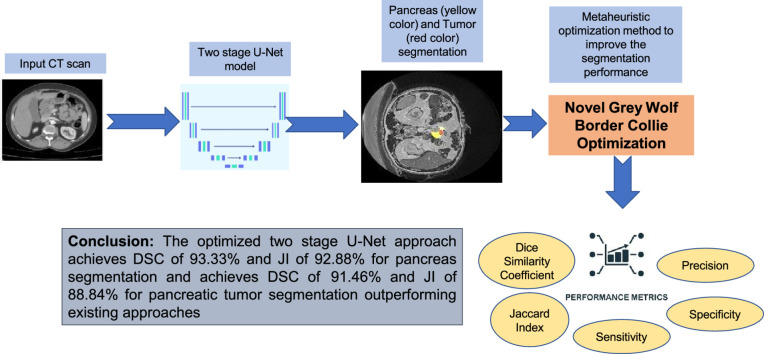

Specifications table

This table provides general information on your method.

**Table:** 

Subject area:	Engineering
More specific subject area:	Medical Imaging
Name of your method:	A two-stage hybrid U-Net model with Grey Wolf Border Collie Optimization (GWBCO) method
Name and reference of original method:	Ronneberger, O., Fischer, P., & Brox, T. (2015). U-net: Convolutional networks for biomedical image segmentation. In Medical image computing and computer-assisted intervention–MICCAI 2015: 18th international conference, Munich, Germany, October 5–9, 2015, proceedings, part III 18 (pp. 234–241). Springer International Publishing.Doi:10.1007/978-3-319-24574-4_28
Resource availability:	Data: http://medicaldecathlon.com/ Software: Pycharm, PyTorch library

## Background

Accurate segmentation of pancreas and pancreatic tumor is vital for accurate cancer diagnosis and prognosis. However, manual segmentation of medical images is quite time consuming and labor intensive. Recently, deep learning-based methods leveraging Convolutional Neural Networks (CNNs) have emerged as a prominent solution to automate the segmentation process. These CNN-based approaches learn from annotated medical images enabling accurate and efficient segmentation of pancreas and tumors. U-Net [1] basically designed for biomedical image segmentation, utilizes a contracting path for context extraction and expanding path for localization aided by skip connections which enhances segmentation accuracy. Furthermore, finetuning of U-Net architecture using various optimization techniques also enhances the segmentation accuracy. While identifying the tumor using U-Net it is crucial for pinpointing the diseased portion, incorporating pancreas segmentation adds significant value by providing a comprehensive anatomical context. Segmenting the entire pancreas improves tumor localization within the organ, supports more effective treatment planning by delineating healthy tissue and surrounding structures and enhances longitudinal monitoring of disease progression. Additionally, it enriches the dataset used for developing and training diagnostic models, leading to more accurate and robust tools. By refining U-Net model through the optimization strategies we aim to provide more reliable segmentation results, contributing to improved diagnostic accuracy and treatment planning for pancreatic tumor.

## Method details

An automated hybrid approach for that segments pancreas and pancreatic tumor is presented in this paper. The hybrid approach consists of a 2D U-Net model and two different metaheuristic optimization methods, namely Grey Wolf Optimization (GWO) [2] and Border Collie Optimization (BCO) [3]. Initially pancreas followed by pancreatic tumor are segmented using U-Net from a CT scan, then optimization is performed to improve the segmentation performance. The optimization approach, Grey Wolf Border Collie Optimization (GWBCO) is developed by combining GWO and BCO.

**Fig. 1:**
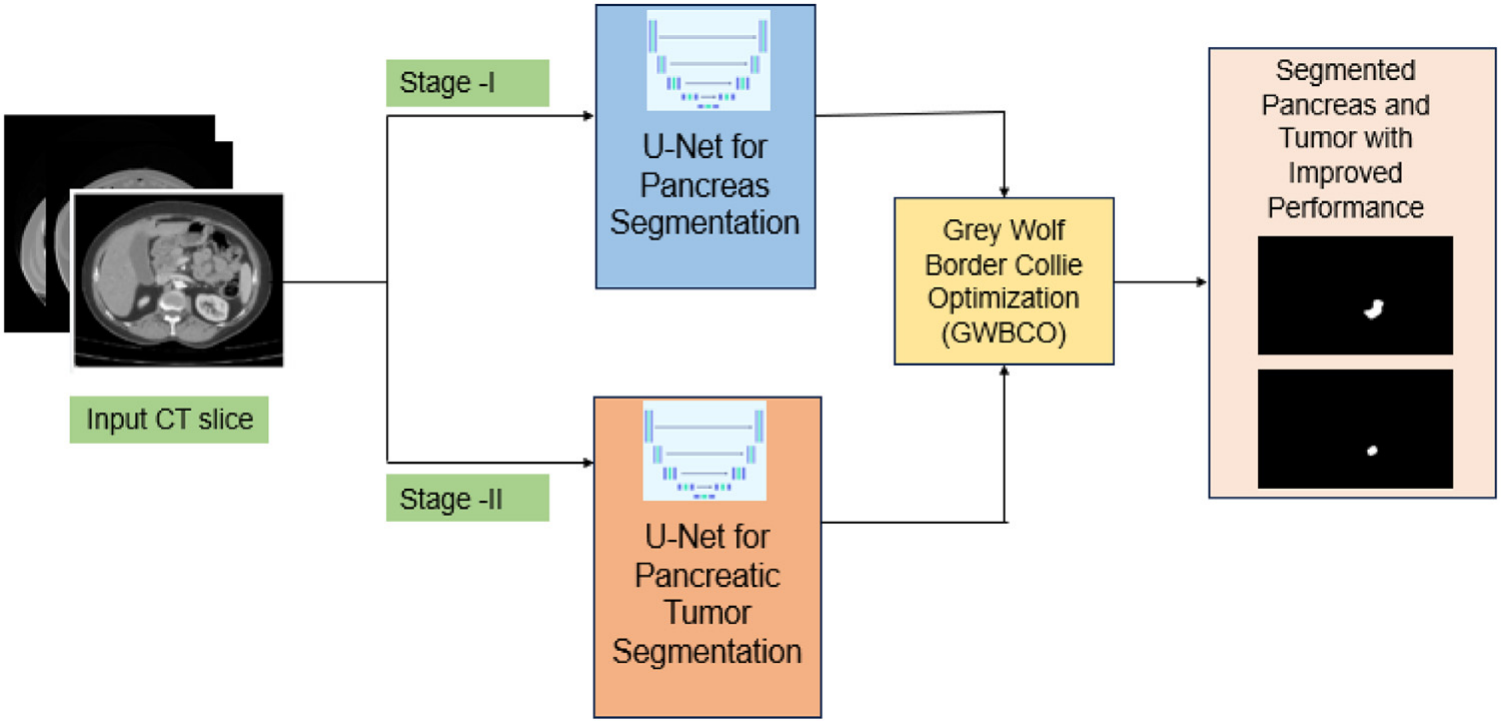
Two-stage segmentation architecture for pancreas and pancreatic tumor.

The proposed model comprises a dual stage cascaded U-Net model integrated with the novel grey wolf Border Collie Optimization method aimed at enhancing the effectiveness of U-Net model. Fig. 1 represents the two-stage framework for segmentation of pancreas and tumor. Stage I performs pancreas segmentation while stage II performs segmentation of pancreatic tumor. The model weights are then optimized using GWBCO method which helps to enhance the segmentation accuracy.

### Two stage cascaded U-Net model

**Fig. 2:**
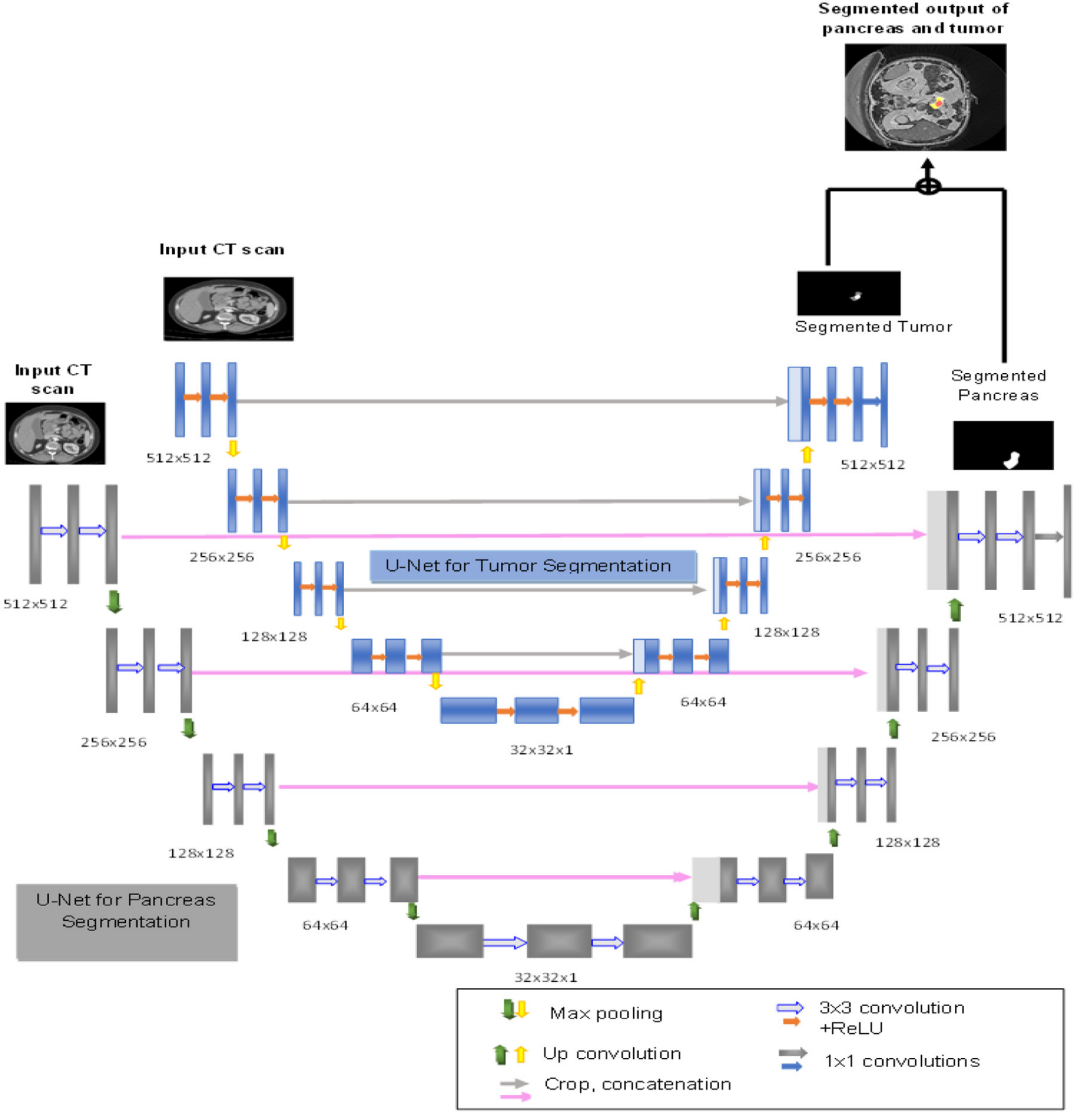
The two stage U-Net model for pancreas and pancreatic tumor segmentation.

U-Net is widely recognized as leading CNN architecture in medical image segmentation tasks that is beneficial for numerous applications in medical imaging including pancreas and pancreatic tumor segmentation. The term U-Net is formulated due to its distinctive “U” like shape comprises of encoder on left side and decoder on the right side. However, the model has certain limitations; one of the common challenges in this model is its susceptibility to handling class imbalance cases in medical imaging datasets where majority of the pixels correspond to non-tumor regions. To tackle these limitations the research introduces a dual-stage cascaded U-Net Model that segments both pancreas and pancreatic tumor from CT scans. Unlike U-Net, dual stage cascaded U-Net model the input image is compressed by the encoder representing in a latent space, while the decoder block reverses the process to restore the input image. These blocks make up the fundamental blocks of U-Net model. First stage network performs pancreas segmentation, and the second stage performs segmentation of pancreatic tumor both from whole CT scan. The two stage segmented outputs are fused to produce a prediction output of pancreas and tumor. The 2D U-Net model represented in Fig. 2, comprises four convolutional blocks in an encoder (contractive path). This encoder consists of 3 × 3 convolutional layers then an activation function and max pooling layer. The 2 × 2 max pooling operation is employed to reduce feature map size required for segmentation process. The 2D CT slice with dimensions of 512 × 512 passes through the series of convolutional blocks; the encoder path extracts the contextual information and reduces the resolution by half. The two stage U-Net contains four decoder units (expanding path) which up-samples the feature map with 2 × 2 up-convolution layer. The feature map from the respective layers in the encoder path is cropped and concatenated to the up-sampled feature map in the decoder path which is followed by the two successive 3 × 3 convolutions, ReLU activation and up convolution layers. The skip connections in the decoder generate better semantic features. After four up-sampling blocks and convolutional layers, the binary image is reconstructed of size 512 × 512. To produce a segmented image additional 1 × 1 convolutional layer is employed in this architecture which generates the final logits. Predicted outputs from both networks are fused to produce the segmented output.

### Optimization

Optimization can be performed during training and post training of the model. Optimization during training refers to adjusting the parameters of the model to minimize or maximize the objective function. The objective function quantifies the variation between model's actual output and predicted output and helps to improve the model performance. Post processing optimization can be used to finetune the model output. In this paper post processing optimization is conducted to enhance segmentation accuracy. Two stage U-Net model generates initial segmentation maps and then these segmentation maps are refined using optimization methods [4].

### Metaheuristic optimization method

The classical optimization approaches struggle to handle complex and nonlinear optimization tasks. To overcome this recently metaheuristic optimization algorithms [5] have become increasingly popular. Metaheuristic optimization algorithms have a higher-level approach to determine the optimal solutions. They are characterized for their wide range of optimization tasks [6]. These methods produce better results with less computational complexity than the classical optimization methods. There are various types of metaheuristic optimization methods such as swarm-based, evolutionary, human-based and physics-based algorithms. This paper focuses on swarm-based metaheuristic algorithms that consist of Grey Wolf Optimization and Border Collie Optimization. The collective behavior of the swarms is replicated in the swarm-based techniques, allowing for effective exploration of complicated problem domains and the finding of optimal solutions [7]. The goal of developing these algorithms is to solve complicated optimization problems that cannot be solved using traditional optimization approaches. These methods are successful for tackling complicated optimization issues, particularly for many hybrid real-world problems. In other words, the primary benefit of meta-heuristic methods is its broad applicability and effectiveness. Meta-heuristic algorithms are specifically designed to tackle complex problems and have recently gained traction in different image analysis applications, notably in the classification and segmentation of tumors [[8], [9], [10], [11]]. The two important features of these algorithms are exploration and exploitation. Various parameters are employed by the conventional optimization algorithms to balance these two characteristics, and help to discover better solutions Classical optimization methods are rarely used these days as it requires further parameter adjustment [12].

### Grey Wolf Border Collie Optimization method for improving segmentation

In this study a new combined metaheuristic optimization algorithm is employed into a two stage U-Net network, consisting Grey Wolf Optimization (GWO) algorithm and Border Collie Optimization (BCO) algorithm. The combination of GWO and BCO algorithm referred as Grey Wolf Border Collie Optimization (GWBCO) algorithm optimally tunes the cascaded U-Net model. GWO is optimization inspired by nature specifically modeling the social behavior observed in grey wolves. GWO helps to enhance the accuracy and convergence of segmentation model. The other optimization strategy integrated with U-Net is the Border Collie Optimization (BCO). The herding characteristics of border collies are employed to perform the optimization. The fusion of U-Net with both GWO and BCO is the key innovation in the proposed segmentation model. This hybrid approach uses the unique capabilities of both the optimization methods to maximize the precision and efficiency in segmentation.

### Grey Wolf Optimization method

Recently, one of the prominent metaheuristic optimization techniques is Grey Wolf Optimization. The name “Grey Wolf Optimization” is derived from the hunting characteristics of the grey wolves. It also consists of hierarchical leadership characteristics. These leadership hierarchies consist of four different types of grey wolves such as alpha, beta, delta and omega characteristics. The optimal solution is achieved by iteratively updating the position of these grey wolves. It consists of various phases such as searching, attacking, hunting and encircling [2]. Recently this optimization method is utilized for various image analysis tasks such as detection, diagnosis, segmentation and classification [13,14]. The integration of GWO helps to improve the effectiveness of segmentation and helps to achieve more accurate diagnosis.

### Border Collie Optimization method

A new swarm-based metaheuristic optimization method is introduced by Dutta et al. [3]. The name “Border Collie Optimization” is acquired from the border collie dogs which perform herding of sheep. Various herding techniques are employed by border collies such as gathering, stalking and eyeing. The main advantage of this optimization is they minimize the parameters of exploitation. The eyeing characteristics and algorithms intrinsic capabilities help to improve the performance of segmentation model. BCO has various benefits such as the ability to avoid local minima exploit and traverse the search space efficiently and improvise the performance. This helps to easily determine the maximum and minimum fitness values.

### Inspiration of Grey Wolf and Border Collie Optimization

The GWBCO algorithm is inspiration from both algorithms, including hunting characteristics of GWO and herding traits of border collies. In the context of pancreas and pancreatic tumor segmentation, GWBCO can be utilized to refine and optimize parameters of segmentation algorithms, enhancing the accuracy of delineating tumor boundaries and ensuring a more precise identification of tumor regions in medical images.

The GWBCO algorithm draws inspiration from the hunting traits of grey wolves within the wolf packs; they follow a hierarchy for hunting. The alpha is known as the leader of the pack who excels in decision-making and dictating the packs, the leaders are not the strongest but they can manage the pack effectively. The secondary leader is known as the beta which respects the primary leader who acts as an advisor, and supports the leaders of the pack. Omega wolves and delta wolves fulfill different roles such as scouts, hunters, and caretakers they contribute to the well-being of the pack [2]. The hunting mechanism is directed by the primary leader. The exploration is made by primary leader and other subordinates follow the leader. The herding traits are inspired by the border Collies also known as herding dogs, which possess remarkable decision-making and herding skills [3]. The dogs follow three herding strategies: gathering, stalking, and eyeing. To direct the sheep to its distinct location the gathering strategy employed flanking and frontward control. The border collies also exhibit the victim selection known as eyeing, which exhibits an intense gaze that exerts psychological pressure on the sheep and encourages them to travel in the right direction.

The standard optimization based on the hunting traits of a grey wolf has limitations such as low convergence speed, minimum solution accuracy, and local optimum problems which limits the model performance. To rectify the local optimum and convergence problem the herding (eyeing) traits of the border collie are integrated into this algorithm which avoids local minima and enhances the convergence capability of the algorithm.

### Solution initialization

In this research, the tunable parameter of U-Net model is optimized using GWBCO algorithm. The solution is initialized as$\;R^{t+1}$.

### Fitness evaluation

A possible solution is fed into the fitness function, which returns a numerical value indicating the solution's effectiveness or fit. A better solution in terms of the objectives being pursued is indicated by a higher fitness value. Lower fitness values, on the other hand, signify inadequate performance. The fitness function for this algorithm is

(1) $$Ft\left(R^{t+1}\right)=DSC\left(R^{t+1}\right)$$

WhereDSC represents the dice similarity co-efficient

Initially, the solution in the search space is written as

(2) $$\;R^{t+1}=R_{rand}+\left(v^{t}-v^{t-1}\right)\left|u-l\right|$$

Where uand ldenotes the upper and lower bound, $R_{rand}$represents the random population, $v^{t}$ denotes current velocity, $v^{t-1}$ represents the previous velocity.The search space (φ) is between 0 and 1. Here two cases are considered encircling phase and observation phase. The highest border is considered as 0.9, if the prey is within the search space i.e., ϕ<0.9 the predator encircles and attacks the prey and updates theirpositions. If the prey is away from the search space i.e., ϕ>0.9the predator searches the prey out of the boundary. Both the cases with mathematical equations are explained below.

Case (i)ϕ<0.9

Encircling phase:

To describe the social structure of wolves, the alpha (α) denotes the best fit solution, following that beta (β) and delta (δ) are the second and third best solution. If solution (grey wolf) finds any prey in the search space φ, the solution encircles the prey. The encircling behavior is represented by the following equation,

(3) $$\;R_{1}=R_{\alpha }-P_{1}\left(\left[Q_{1}.R_{\alpha }-R_{t}\right]\right)$$

(4) $$\;R_{1}=R_{\alpha }\left(1-P_{1}Q_{1}\right)+P_{1}R_{t}$$

Similarly, the moment based on the second and third-best solutions is updated as

(5) $$R_{2}=R_{\beta }\left(1-P_{2}Q_{2}\right)+P_{2}R_{t}$$

(6) $$R_{3}=R_{\delta }\left(1-P_{3}Q_{3}\right)+P_{3}R_{t}$$

Where the coefficient vectors PandQare computed as

(7) $$P=2pr_{1}-p$$

(8) $$Q=2r_{2}$$

Where the$P_{1},$ $P_{2},$and $P_{3}$ as well as $Q_{1,\;}Q_{2},$ and $Q_{3}$ varies based on $r_{1}$and$r_{2}$, Components of plinearly decreased from 2 to 0 during iterations, $r_{1}\;$and$\;r_{2}\;$are random numbers which are represented as

(9) $$\left(1,0\right)\in \left\{ \begin{array}{c} r_{1}=R_{t}e^{\frac{t-1}{t_{max}}}\\ r_{2}=R_{t}e^{\frac{t}{t_{max}}} \end{array}\right.$$

Where tdenotes the current iteration and $t_{max\;}$denotes maximum iteration. Using above equations, the solution's position is adjusted according to the position of prey.

### Attacking phase

After encircling, the solution proceeds to hunt and the solution finishes the hunt and captures the prey. In every iteration, position of remaining solutions (ω)is updated based on the positions of α,β, and δ. Since these three has better knowledge about the prey's location and is mathematically represented as,

(10) $$\;R^{t+1}=\frac{R_{1}+R_{2}+R_{3}}{3}\;$$

(11) $$R^{t+1}=\frac{R_{\alpha }\left(1-P_{1}Q_{1}\right)+P_{1}R_{t}+R_{\beta }\left(1-P_{2}Q_{2}\right)+P_{2}R_{t}+R_{\delta }\left(1-P_{3}Q_{3}\right)+P_{3}R_{t}}{3}$$

By adjusting the value of PandQ different places around the best agent can be reached to the current position. But this may stuck in the local optimum problem.

To prevent the local optimum problem solutions, find the prey based on its velocity, acceleration, and time using the eyeing characteristics, which is described as

(12) $$R_{prey}^{t+1}=V_{prey}^{\left(t+1\right)}\times Time^{\left(t+1\right)}-\frac{1}{2}A_{prey}^{\left(t+1\right)}\times Tim{e^{2}}^{\left(t+1\right)}$$

Where the velocity of the prey is denoted as$V_{prey}^{\left(t+1\right)}$and $\;A_{prey}^{\left(t+1\right)}$represents the acceleration of prey. The velocity of the prey was updated using the following equation

(13) $$\;V_{prey}^{\left(t+1\right)}=\sqrt{V^{{\left(t+1\right)}^{2}}-2A^{\left(t+1\right)}*\left|R^{t}-R^{t-1}\right|}$$

Where$|R^{t}-R^{t-1}|$ represents the distance between the positions from the current to previous iteration, the acceleration is updated as

(14) $$A^{\left(t+1\right)}=\frac{V^{\left(t+1\right)}-V^{t}}{Time^{\left(t+1\right)}}$$

Where $V^{\left(t+1\right)}-V^{t}$ represents the change in velocity

(15) $$\begin{array}{ccc} R^{t+1} & = & \frac{R_{\alpha }\left(1-P_{1}Q_{1}\right)+P_{1}R_{t}+R_{\beta }\left(1-P_{2}Q_{2}\right)+P_{2}R_{t}+R_{\delta }\left(1-P_{3}Q_{3}\right)+P_{3}R_{t}}{3}\\ & & +\;\rho_{f}\left(V_{prey}^{\left(t+1\right)}\times Time^{\left(t+1\right)}-\frac{1}{2}A_{prey}^{\left(t+1\right)}\times Tim{e^{2}}^{\left(t+1\right)}\right) \end{array}$$

Where $\rho_{f}$represents the probability factor which is equal to 1

The best position of the solution is updated using the following equation

(16) $$R^{t+1}=\frac{R_{\alpha }\left(1-P_{1}Q_{1}\right)+R_{\beta }\left(1-P_{2}Q_{2}\right)+R_{\delta }\left(1-P_{3}Q_{3}\right)+R_{t}\left(P_{1}+P_{2}+P_{3}\right)+3V_{prey}^{\left(t+1\right)}\times Time^{\left(t+1\right)}-\frac{1}{2}A_{prey}^{\left(t+1\right)}\times Tim{e^{2}}^{\left(t+1\right)}}{3}$$

Thus, the above equation with the eyeing technique must improve convergence speed and local optimum problem.

Case (ii)ϕ>0.9

Observation Phase

If the solution does not find any prey within the search space, the solution searches prey outside the boundary and update the location of the solution based on prey's location.

(17) $$R^{t+1}=R_{g}+K_{m}\left(R_{prey}-R_{t}\right)$$

**Fig. 3:**
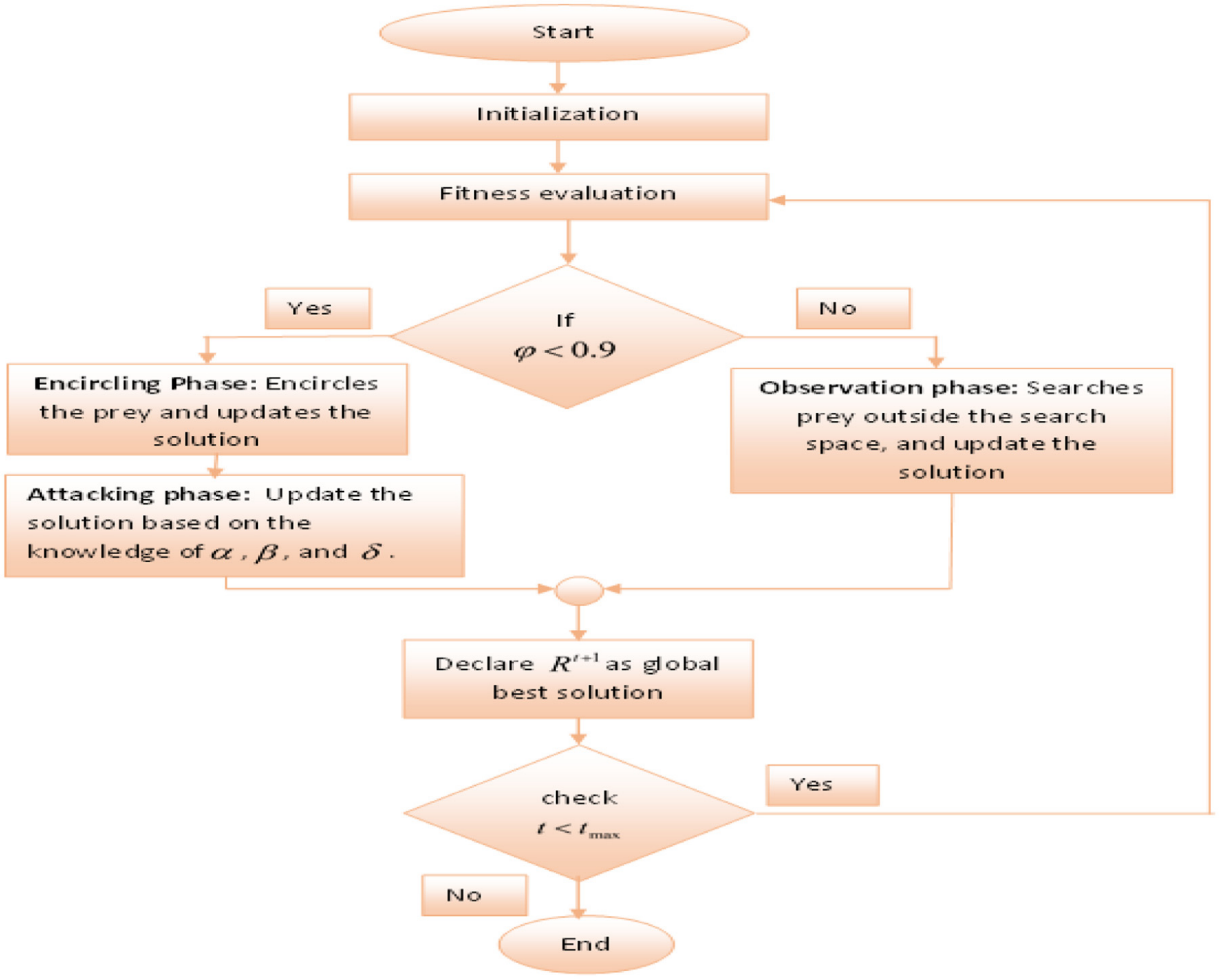
The flowchart of the GWBCO algorithm.

Where, $R_{g}$ indicates the global best solution, $K_{m}$ is the movement factor, $R_{prey}$ is prey's position, $R_{t}$ is solution's current position. Fig. 3 represents the flowchart of the GWBCO algorithm. The GWBCO algorithm is based on different phases which help in obtaining the best solution through iterative refinement. Each phase contributes to optimizing the parameters and finetuning the U-Net architecture for improved segmentation accuracy.

### GWBCO in parameter optimization

The primary goal of GWBCO is to adjust U-Nets weights and biases iteratively, helping the model to yield a better segmentation. Basically, the parameters fine-tuned by this GWBCO optimization are weights and biases of U-Net model. Instead of using traditional gradient descent, GWBCO adjusts the weights and biases by simulating the behavior of wolves and border collies. In this analogy sheep represents the set of potential solutions i.e. different configurations of U-Net parameters and herding represents the process of guiding these solutions towards an optimal configuration that improves the performance of U-Net. Concurrently, the hunting behavior of grey wolves is employed to focus on and exploit the most promising candidate solutions narrowing down the search space efficiently.

The combination of herding and hunting in GWBCO ensures that the optimization process is both explorative and exploitative, allowing for a thorough search of the parameter space while steadily driving the candidate solutions towards an optimal set of parameters. This dual approach ultimately leads to a refined U-Net that performs better on the metrics such as Dice similarity coefficient, Jaccard index etc.

**Fig. 4:**
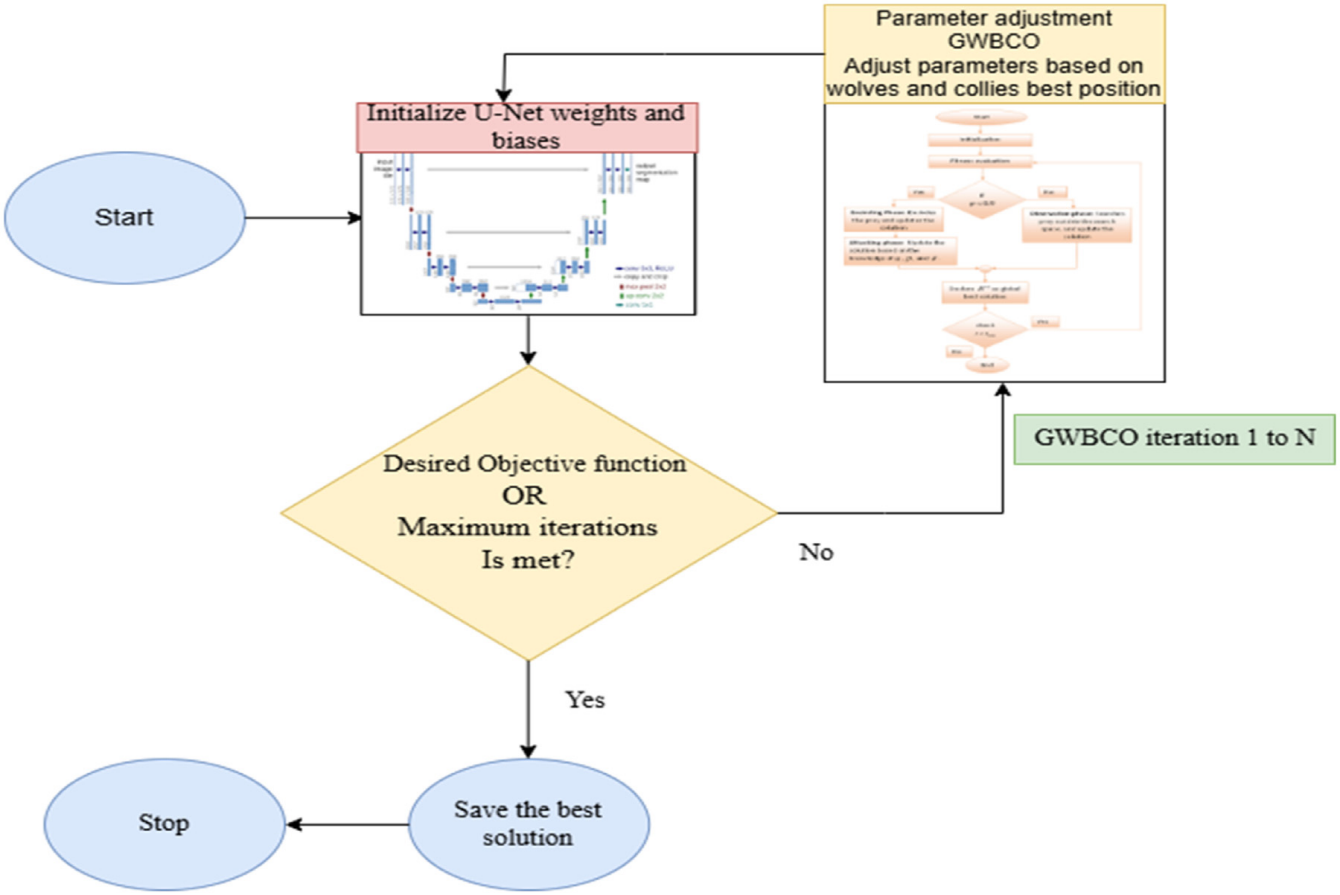
Relationship between GWBCO and U-Net.

Fig. 4 represents the relationship between GWBCO and U-Net weights and biases. Initialize the U-Net with random weights and biases. These parameters will be updated iteratively through GWBCO algorithm to improve the model's performance on segmentation task. In each iteration, the GWBCO algorithm evaluates the U-Net model's performance by calculating objective function. Based on this, the algorithm adjusts the positions of wolves and collies to move towards better position. These adjusted positions are used to update the U-Net model's weight and biases. The iteration continues until either the desired number of iterations is reached or the objective function is achieved. After each iteration the algorithm checks whether the desired function value has been met else algorithm proceeds with further iteration continuing to adjust U-Net's parameters. If the objective function is not yet achieved, GWBCO continues to adjust the U-Net's weights and biases based on the best position in the current iteration. Once the objective function is met or the maximum number of iterations is reached, the optimization process stops. The best U-Net model parameters found by GWBCO during the iterations are saved as the final solution. The optimization process concludes, and the optimized U-Net model is ready for further evaluation.

## Method validation

### Dataset

The dataset selected for segmentation is publicly available, Medical Segmentation Decathlon (MSD) Challenge dataset comprises of 420 portal venous phase CT scans from which 282 are used for training and 139 for testing. The dataset consists of whole abdominal CT, segmented mask for pancreas and pancreatic tumor. CT scan size varies from 512 × 512 x l where (l ranges from 37 to 751) with a thickness of 2.5 mm. The dataset was acquired from a cancer center namely Memorial Sloan Kettering Cancer Center (MSKCC), New York, US [15]. Preprocessing of CT scans is essential in medical imaging to ensure clarity and accuracy. In this work min-max normalization is applied to prepare the data for training and testing. The CT values are normalized to the range of [0,1]. This involves scaling the pixel values based on minimum and maximum values in each image, allowing for uniformity across the dataset. By implementing this normalization step, we enhance the quality of the input data, thereby facilitating more effective learning and improving the model's performance in accurately segmenting pancreatic structures.

**Fig. 5:**
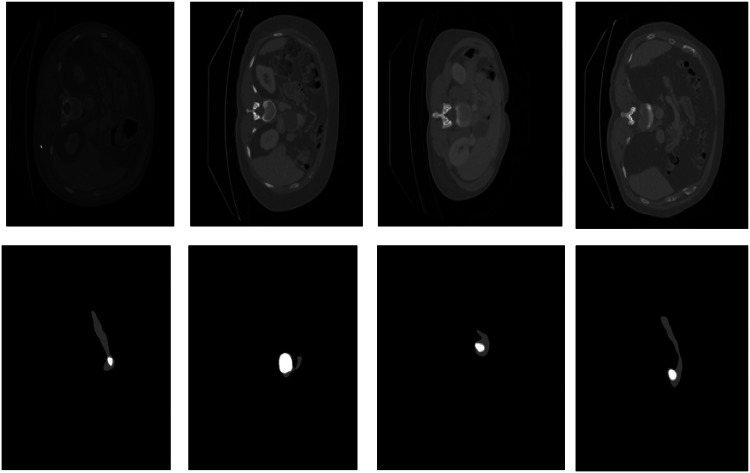
The first row shows sample images and second row represents corresponding mask of pancreas in grey and pancreatic tumor in white from medical segmentation decathlon dataset*Model Parameters and Performance Metrics*.

Fig. 5 shows the sample images of a CT slice and mask of pancreas and pancreatic tumor from the dataset.

Hybrid algorithms combine many optimization approaches or structures to increase overall performance. Combining the grey wolf and border collie algorithm with U-Net model creates a hybrid algorithm. The U-Net model parameters are optimized by GWBCO algorithm. The parameters of U-Net are considered as decision variables and the optimal values are achieved by GWBCO. Initially training dataset consisted of 40 % and testing dataset of 60 %. For every iteration the size of training set is increased by 10 % and testing set is reduced by 10 %. This is done to ensure that the model trains on as much diverse data as possible while still maintaining a consistent way to evaluate its performance. This helps to avoid overfitting. The model undergoes training for 500 epochs. Performance of the model is evaluated after every 100 epochs. Subsequently the GWBCO optimization algorithm assist the U-Net network in determining the optimal hyperparameters for the architecture. The iterative process continues until it meets the stop condition, defined as the iterations being less than a predetermined limit, is fulfilled. Once this stop condition is met, the optimal values are assigned to the U-Net design. The segmentation results, including those of test data, are then displayed. Table 1 shows the model parameters and system requirements of U-Net model.

**Table 1:** Hyperparameters and system requirements of U-Net model.

Parameter	Value
Initial learning rate	0.0001
Feature map size	8
Batch size	32
Epochs	500
Convolution kernel size	3 × 3
Python	3.7
RAM	128GB

The GWBCO algorithm also needs to establish with specific tuning hyperparameters. To commence optimization with GWBCO it is necessary to define specific tuning hyperparameters. The population size is 50 and termination condition is set to 50 iterations. In every iteration, the optimization generates position and fitness when the optimization gives the best fitness the corresponding solution will be taken and replaced into the model weights.

### Performance metrics

The performance parameters use to evaluate the model are Dice similarity coefficient (DSC), Jaccard Index (JI), sensitivity, specificity and precision.

Dice Similarity Coefficient (DSC): It is the frequently employed similarity parameter for analysing the segmentation of medical images. It is also called as Dice coefficient or Sørensen-Dice coefficient. This parameter is mostly employed to measure the resemblance between two masks i.e. the ground truth mask and the segmented mask. The DSC ranges between 0 and 1, where 0 represents no overlap between the two masks and 1 represents perfect overlap [16]. The formula to calculate DSC is:

(18) $$DSC=\;\frac{2\;\times \;\left|\;P\;\cap Q\;\right|}{\left|P\right|+\left|Q\right|}$$

where P represents ground truth and Q represents predicted mask.

Jaccard Index (JI): This is the second most commonly employed parameter for evaluating the similarity among ground truth and segmented mask. JI is also known as Intersection Over Union (IOU). It calculates similarity by computing the ratio of intersection with union of both the masks. Similar to DSC it ranges between 0 and 1 representing no overlap and perfect overlap [17]. The formula to calculate JI is:

(19) $$JI=\;\frac{\left|P\;\cap Q\right|}{\left|P\;\cup Q\right|}$$

Precision: Precision is defined as the truly positive voxels in the segmentation. Greater the precision value lesser the false positive predictions that results in better segmentation results. It is defined as:

(20) $$Precision=\;\frac{TP}{TP+FP}$$

Where TP (True Positive) indicates the number of voxels accurately recognized as part of pancreas or pancreatic tumor in segmentation mask and ground truth and FP (False Positive) represents the number of voxels mistakenly recognized as part of pancreas or pancreatic tumor in the segmented mask but are not part of the pancreas or pancreatic tumor in the ground truth [18].

Sensitivity: It is the model's ability to accurately identify positive regions (i.e. tumor or pancreas) in the image. It is defined as:

(21) $$Sensitivity=\;\frac{TP}{TP+FN}$$

Where TP (True Positive) represents the positive pixels identified by the model and FN (False Negative) represents the positive pixels that are mistakenly classified as negative by the model [19].

Specificity: It is the model's ability to accurately identify the regions that does not consist of pancreas or tumor mask.

(22) $$Specificity=\;\frac{TN}{TN+FP\;}$$

Where TN (True Negative) represents the negative pixels identified by the model and FP (False Positive) is represents the negative pixels that are mistakenly classified as positive by the model [19]. Both sensitivity and specificity are opposite to each other as sensitivity detects the positive pixels and specificity detects negative pixels.

## Results and discussion

In this section we present the qualitative outcomes achieved by U-Net GWBCO model and provide a comprehensive comparison with existing methods. Additionally, we conduct ablation studies to assess the contribution of each component within the framework, including U-Net, U-Net GWO, U-Net BCO and the proposed U-Net GWBCO. DSC, JI, sensitivity, specificity and precision are employed for performance evaluation.

## Performance analysis of pancreas segmentation

**Fig. 6:**
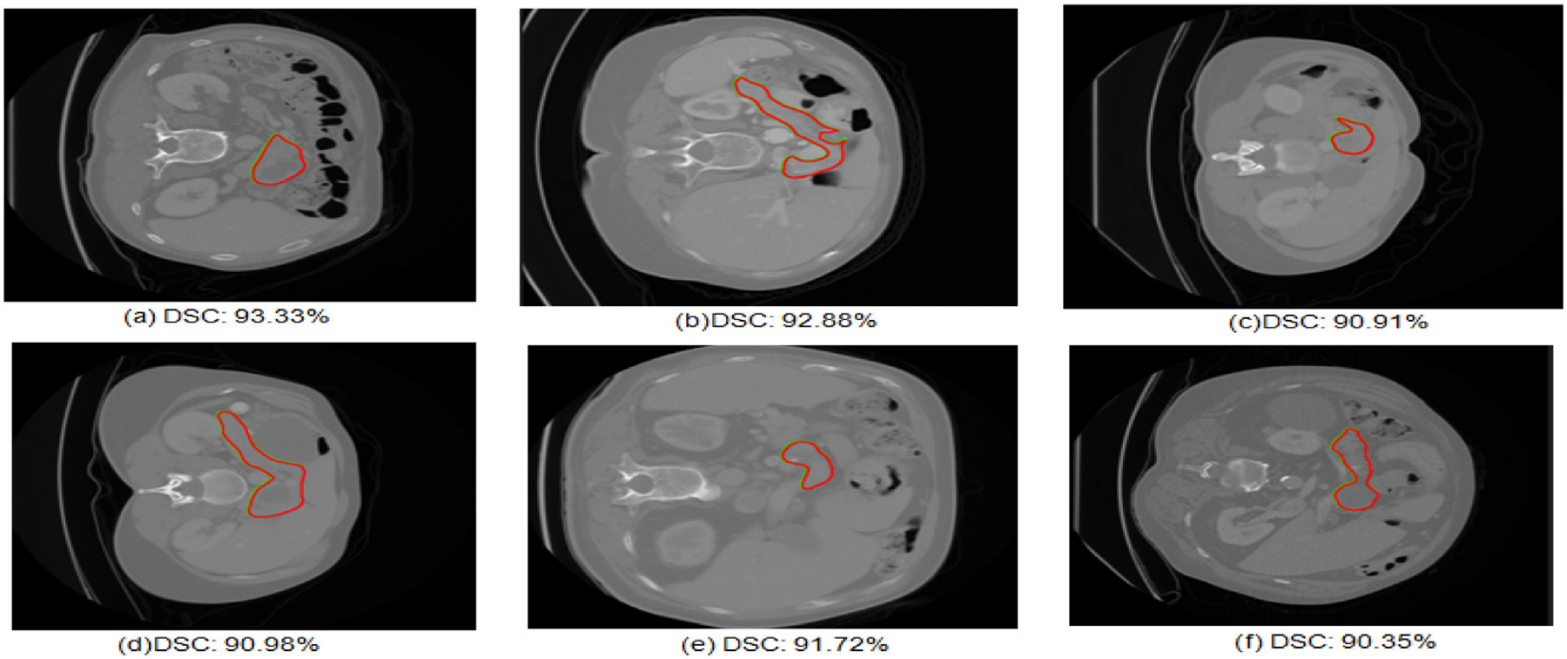
Examples of pancreas segmentation using U-Net GWBCO model. Green contour shows the ground truth and red shows the segmented pancreas.

The analysis of qualitative results and quantitative results can be observed in Fig. 6 and Table 2. Fig. 5 shows some examples of pancreas segmentation results with a comparative overlap of ground truth and segmented output by U-Net GWBCO model. The green contour is ground truth and red contour indicates the segmented pancreas by proposed model. DSC is above 90 % for pancreas segmentation.

**Table 2:** Performance evaluation of pancreas segmentation with the state-of-the-art methods on Medical Segmentation Decathlon (MSD) dataset.

Method	Dataset	Performance metrics (%)				
		DSC	JI	Sensitivity	Specificity	Precision
MAD-UNet, 2021 [20]	MSD	88.52 ± 3.77	79.42 ± 5.82	N/A	89.66 ± 4.62	N/A
Recurrent U-Net, 2021 [21]	MSD	85.65	N/A	N/A	N/A	N/A
RTUNet,2023 [22]	MSD	86.25 ± 4.52	N/A	N/A	N/A	N/A
TD-Net, 2023 [23]	MSD	91.22 ± 1.37	N/A	91.35 ± 1.63	N/A	**93.22 ± 2.79**
M3BUNet, 2023 [24]	MSD	88.60 ± 1.48	79.9 ± 2.19	87.20 ± 2.83	**95.71 ± 0.78**	90.76 ± 1.42
ResDAC—Net,2024 [25]	MSD	69.15	58.76	72.31	N/A	81.69
AX-UNet,2022 [26]	MSD	85.9 ± 5.1	77.9 ± 3.4	86.3 ± 5.1	N/A	93.1 ± 6.9
MoNet,2021 [27]	MSD	74	68.9	N/A	N/A	N/A
UCFilTransNet,2023 [28]	MSD	82.83	72.84	N/A	N/A	N/A
nnU-Net,2019 [34]	MSD	82.00	N/A	N/A	N/A	N/A
**U-Net-GWBCO (Ours)**	MSD	**93.33**	**92.88**	**91.99**	91.84	91.36

The variable size and shape of pancreas of different patients depicts it is quite difficult for radiologists to appropriately segment the pancreas. The quantitative results are improvised due to the GWBCO algorithm. The quantitative results of pancreas segmentation and its comparison with existing methods is represented in Table 2.

The results represented Table 2 show that our model proves to be more efficient in the various performance metrics. Furthermore, our model outperforms the well-known nnU-Net model for pancreas segmentation.Dice Similarity Coefficient (DSC), a critical statistic to calculate segmentation similarity, is especially significant. The obtained DSC, JI, Sensitivity values show improved performance. This demonstrates our model's strong ability to effectively define and represent the complexities of pancreatic structures in CT images.

### Performance analysis of pancreatic tumor segmentation

**Fig. 7:**
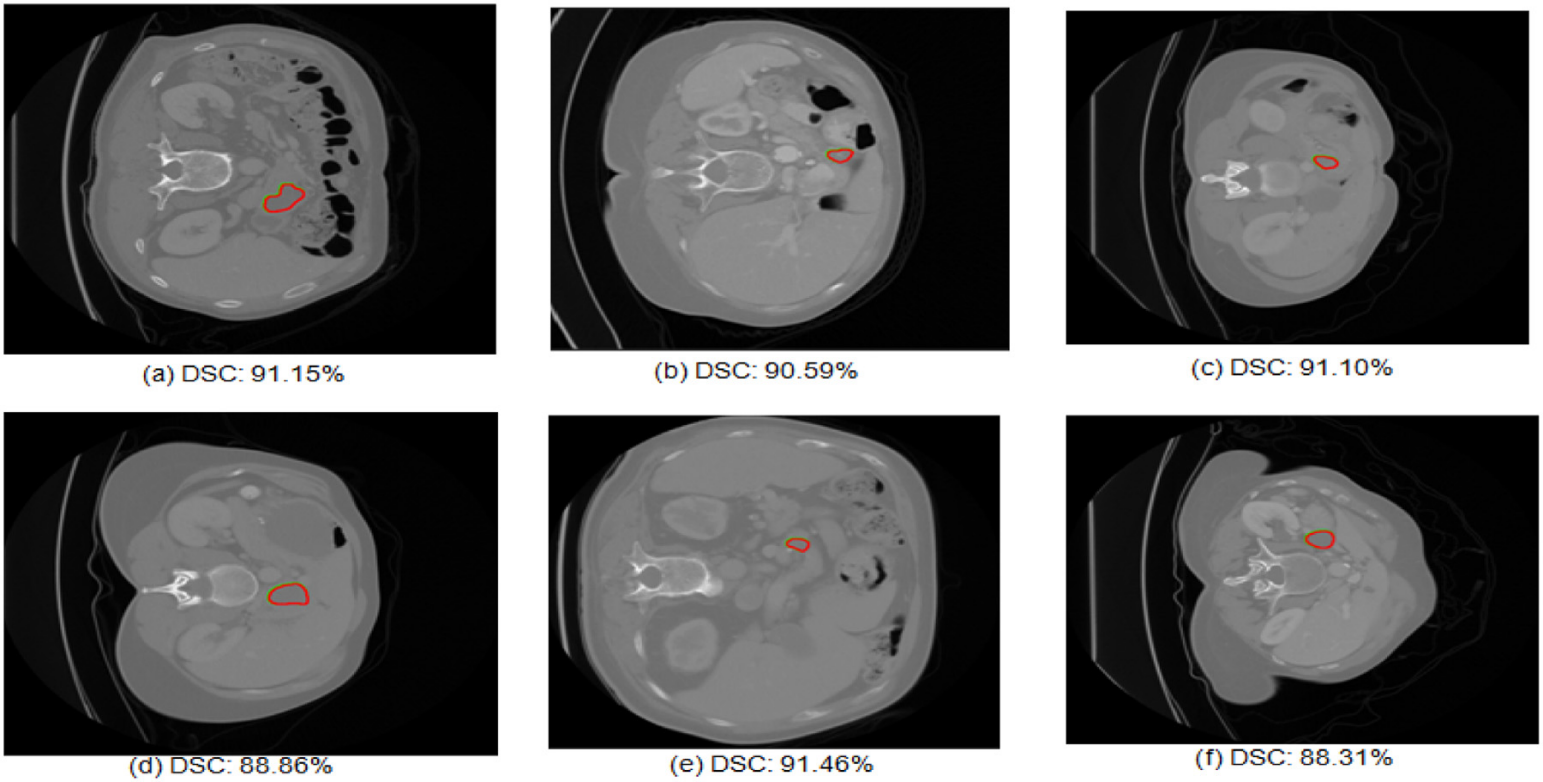
Examples of pancreatic tumor segmentation using U-Net GWBCO model. Green contour shows the ground truth and red shows the segmented pancreatic tumor.

This section presents performance analysis of pancreatic tumor segmentation and also compares the quantitative results with the existing methods. The U-Net GWBCO model for pancreatic tumor segmentation achieves noteworthy performance. Fig. 7 shows the input image given to the second stage of U-Net GWBCO model, corresponding ground truth and segmented mask of pancreatic tumor.

The sample images presented in Fig. 6 illustrate tumors of small size, suggesting the robustness of the model in accurately detecting and delineating even subtle tumor features. This capability underscores the potential clinical utility of the proposed model for early detection and precise characterization of pancreatic tumors, particularly those at an initial stage.

Table 3 depicts the performance evaluation of pancreatic tumor segmentation. All the methods compared in Table 2, Table 3, were evaluated using the same MSD dataset. This ensures that the results are directly comparable, as any potential differences in performance are attributed to the methods themselves rather than variations in datasets. By maintaining a consistent dataset, we provide a fair and standardized comparison across all approaches. The U-Net GWBCO model achieves remarkable results for DSC, JI and other parameters. The overall performance of DSC is increased by 10 % than the MSCA-U-Net model. Moreover, our model outperforms the nnU-Net model in both DSC and sensitivity for pancreatic tumor segmentation. This proves that U-Net model with optimization improves performance than the previous state-of-the-art methods.

**Table 3:** Performance evaluation of pancreatic tumor segmentation with the state of art methods on Medical Segmentation Decathlon (MSD) dataset.

Method	Dataset	Performance metrics (%)				
		DSC	JI	Sensitivity	Specificity	Precision
Temperature guided network, 2023 [29]	MSD	59.16 ± 28.12	79.42 ± 5.82	N/A	89.66 ± 4.62	N/A
TGPFN, 2023 [30] MSCA-U-Net, 2023 [31] SAR, 2021 [32] TAU-Net, 2021 [33] nnU-Net,2019 [34]	MSD MSD MSD MSD MSD	73.93 80.12 33.92 ± 3.00 60.6 54.64	N/A N/A N/A N/A N/A	N/A N/A N/A 78.0 ± 9.0 72.68	N/A N/A N/A N/A N/A	N/A N/A N/A 57.8 ± 23.0 N/A
**U-Net-GWBCO (Ours)**	**MSD**	**91.46**	**88.84**	**88.74**	**97.25**	**86.29**

**Fig. 8:**
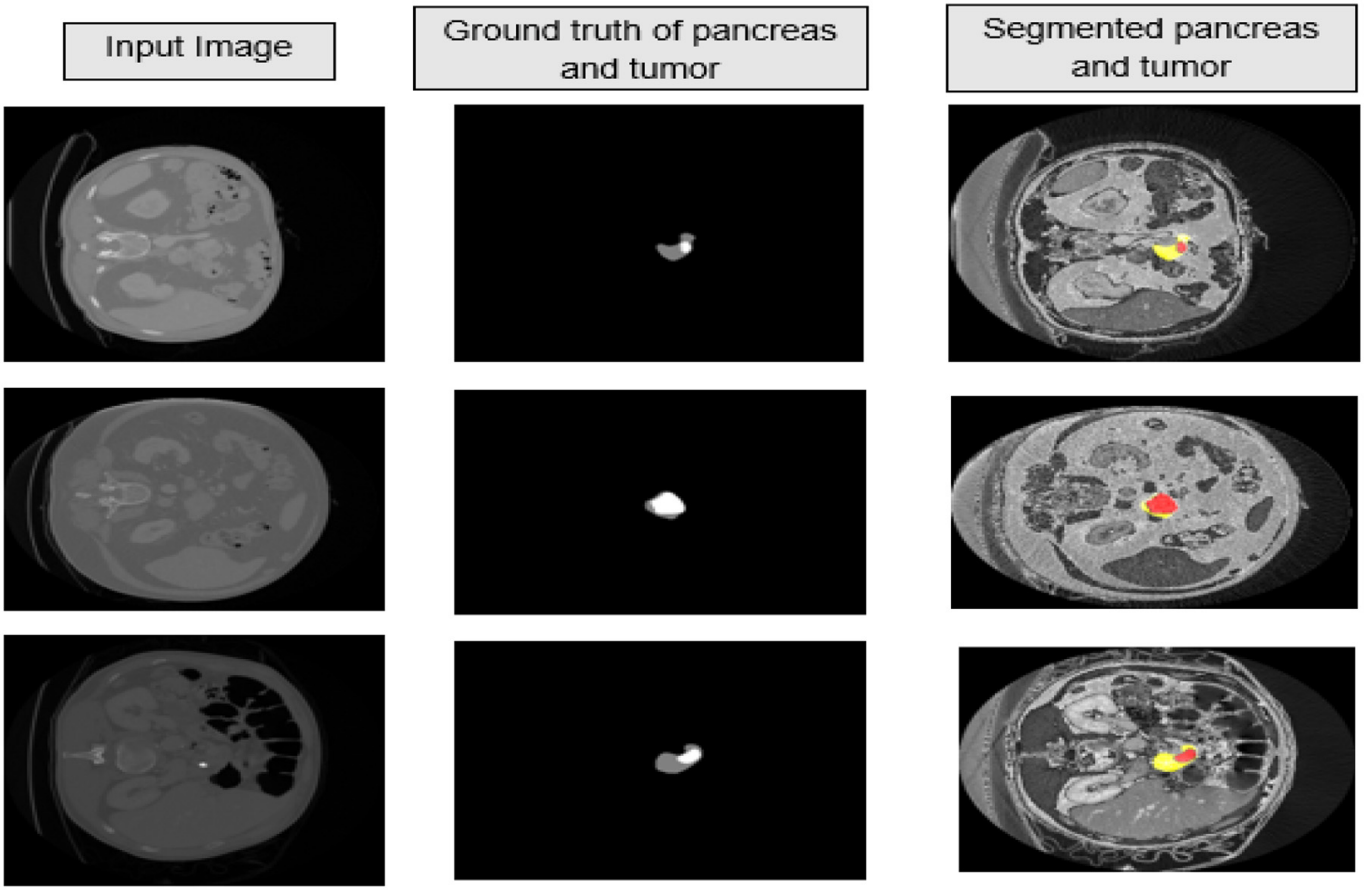
The first column represents three different sample 2D CT slices, second column represents corresponding ground truth of pancreas and pancreatic tumor and third column represents segmented pancreas and pancreatic tumor using proposed model.

Fig. 8 represents the segmented pancreas and pancreatic with pancreas in yellow and tumor in red colour similarly for ground truth the grey colour represents pancreas and the white colour represents the tumor. The quantitative and qualitative results illustrate the effectiveness of U-Net GWBCO model in segmentation of pancreatic tumor.

### Ablation studies

To demonstrate the effectiveness of the proposed U-Net GWBCO model, ablation experiments were performed on MSD dataset with DSC as an evaluation metric. The entire model was divided into sub-frameworks and then compared with the full U-Net GWBCO model. The base U-Net model serves as the foundation, representing the core architectures without any optimization enhancements.

### Base U-Net model

Training the MSD dataset on the base U-Net model without any optimization yields a reasonable segmentation mask, but quantitative results are suboptimal. For pancreas segmentation the DSC achieved is 75 % and for tumor it is approximately 57 % significantly lower than the optimized model. These results, though reasonable, indicate the limitations of the base model in capturing complex features, especially in the challenging task of tumor segmentation.

### U-Net with Grey Wolf Optimization (U-Net GWO)

To improve the performance of the base U-Net model, we integrated the Grey Wolf Optimization algorithm. This optimization technique is designed to observe the model's ability to get optimal results. However, the integration of GWO led to mixed results. The DSC for pancreas segmentation slightly decreased to 69 % while the tumor segmentation improved to 74 %. This reduction in pancreas segmentation accuracy suggests that GWO may not be fully compatible with the base U-Net model for this specific task.

### U-Net with Border Collie Optimization (U-Net BCO)

Replacing the grey wolf optimizer with the Border Collie Optimization method, we observed improved performance for pancreas segmentation with a DSC of 84 %, but the tumor segmentation achieves 62 %. Although this combination performs better than the U-Net GWO for pancreas segmentation, it still falls short in tumor segmentation.

### U-Net with grey wolf Border Collie Optimization (U-Net GWBCO)

Finally, combining both optimization techniques yield significant improvement in both visual and quantitative results for pancreas and pancreatic tumor segmentation. This integration outperforms all the three frameworks by achieving DSC in the range of 93 % and 91 %for pancreas and tumor segmentation.

**Fig. 9:**
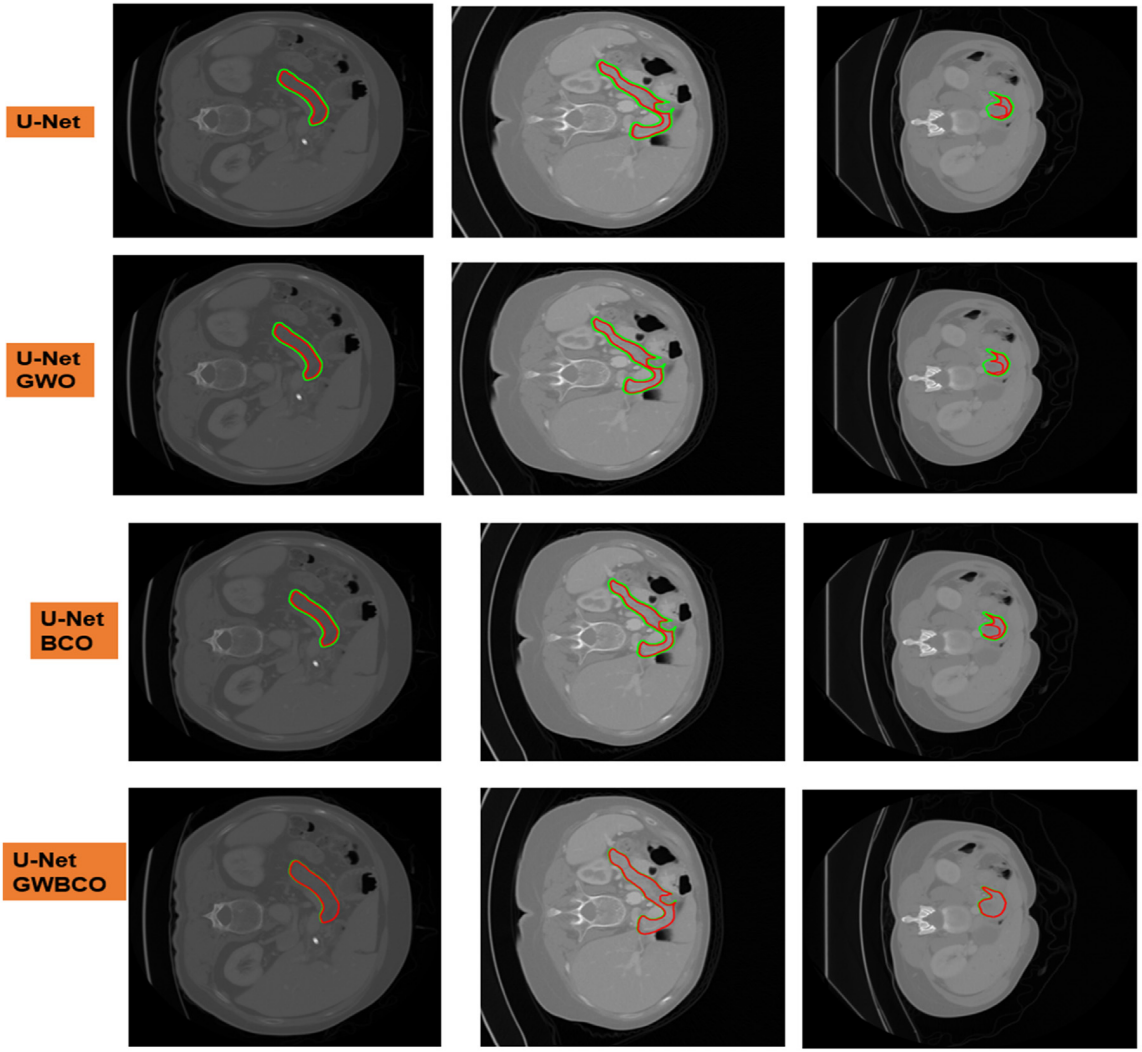
Visual comparison of pancreas segmentation results by U-Net, U-Net GWO, U-Net BCO, and U-Net GWBCO.

**Fig. 10:**
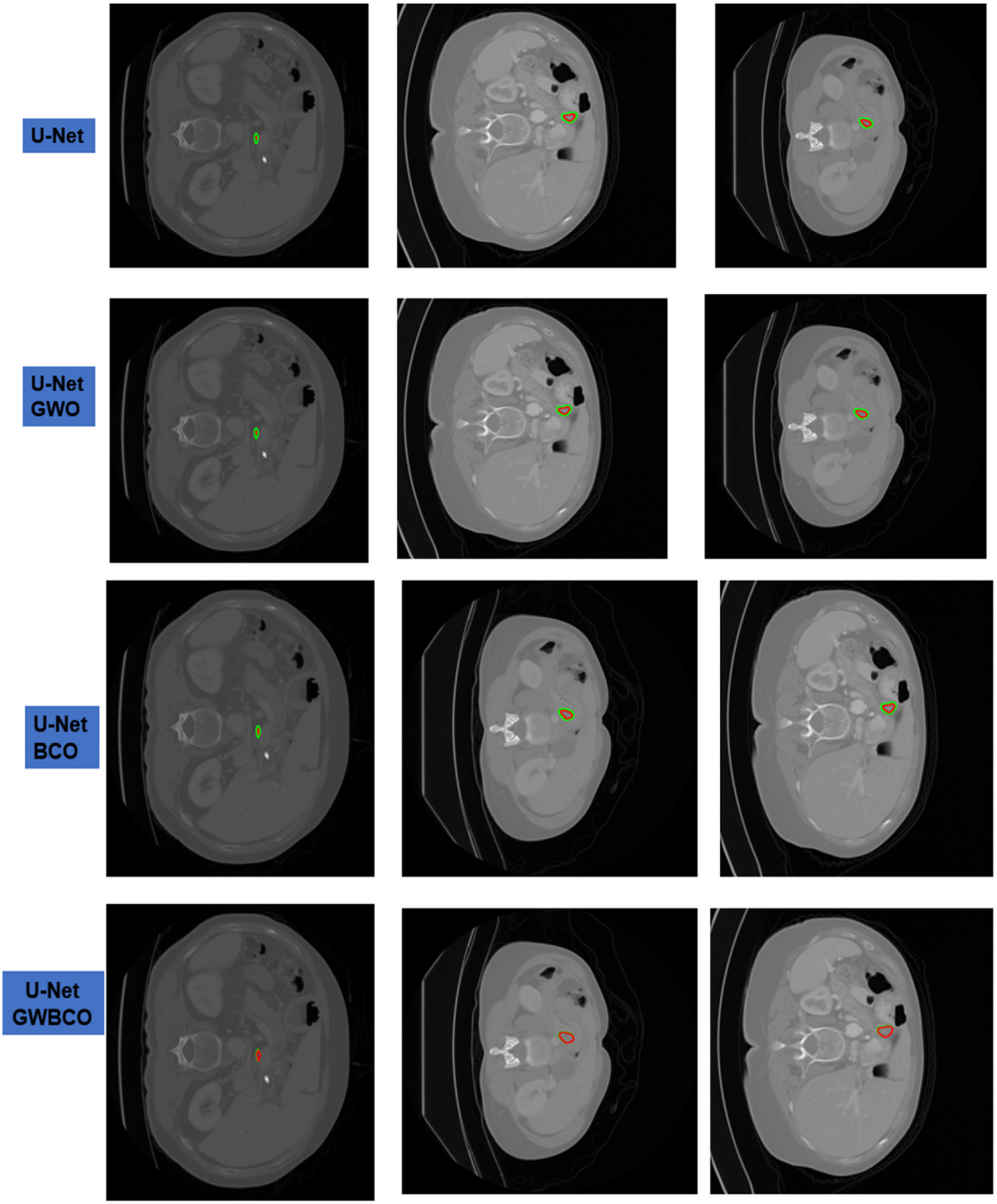
Visual comparison of pancreatic tumor segmentation results by U-Net, U-Net GWO, U-Net BCO, and U-Net GWBCO.

The sample visual qualitative results from these ablation studies are detailed in Fig. 9, Fig. 10. The green colour contour represents the ground truth and red colour contour represents the segmented mask by the model The first three rows represent the U-Net, U-Net GWO, U-Net BCO are not well aligned with ground truth and segmented mask. However, in the U-Net GWBCO model, the alignment is significantly improved, demonstrating the superior performance of this combined optimization approach. The quantitative evaluations of the ablation studies are highlighted in Table 4. The U-Net GWBCO model, with its combined optimizations, consistently outperforms the individual components. This analysis underscores the effectiveness of each optimization technique and validates the proposed model's improvements over standard and individually optimized versions.

**Table 4:** Ablation experiments for pancreas and tumor segmentation using baseline U-Net, U-Net GWO, U-Net BCO, nnU-Net and U-Net GWBCO.

Model	Dataset (MSD)	DSC	JI	Sensitivity	Specificity	Precision
U-Net	Pancreas	75.34	79.84	87.15	79.65	81.00
	Tumor	57.72	71.68	58.17	89.92	57.67
U-Net GWO	Pancreas	69.84	77.97	89.76	85.04	74.49
	Tumor	74.98	66.24	68.97	81.90	59.98
U-Net BCO	Pancreas	84.02	75.80	86.52	73.26	74.05
	Tumor	62.25	64.20	74.19	66.81	69.26
U-Net GWBCO	Pancreas	93.33	92.88	91.99	91.84	91.36
	Tumor	91.46	88.84	88.74	87.25	86.29

Moreover, the results highlight the significance of combining GWO and BCO, as each method addresses specific weaknesses of the other, resulting in a more balanced and effective optimization strategy. The enhanced performance in both pancreas and tumor segmentation underscores the versatility of the U-Net GWBCO model, making it well-suited for handling the variability and complexity of medical images. This approach also suggests potential for application to other medical imaging tasks where both precision and adaptability are crucial. The successful integration of these optimization techniques demonstrates a promising direction for future research in optimizing deep learning models for more accurate and reliable medical image analysis.

## Limitations

**Fig. 11:**
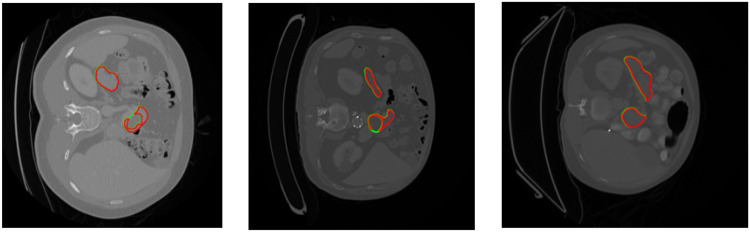
Examples of false positive results by U-Net GWBCO model.

The model exhibits robust performance across various pancreas sizes, achieving better results even for tumors but in some cases the model gives false results. This is because of the class imbalance problem as medical data have large imbalance between background and region of interest. Fig. 11 represents the false positive results of both pancreas and pancreatic tumor generated by model. The other cause of false positive can be due to absence of post processing techniques. Some of the post processing techniques include use of vision transformers and conditional random fields to improve the false positive results. We will try to overcome the limitations in future research.

## Conclusion

The proposed work presents the automated pancreas and pancreatic tumor segmentation, employing a novel two stage U-Net model with GWBCO method. Significant improvements are achieved in performance metrics by integrating U-Net's powerful segmentation capabilities with grey wolf and Border Collie Optimization process. The U-Net-GWBCO framework outperforms existing segmentation algorithms by offering exact delineation of anatomical features and target regions in medical images. The performance metrics demonstrate notable advancements in segmentation. Achieving DSC, JI, sensitivity, specificity, and precision rates surpassing 91 % to 93 % for pancreas segmentation and between 86 % and 91 % for pancreatic tumor segmentation signify significant progress over existing methods. These outcomes underscore the efficacy and potential of U-Net GWBCO approach in addressing the challenges of analyzing medical images. The integration of optimization methods helps to achieve optimal results. Our study helps to advance medical image analysis by providing effective segmentation tool for accurate diagnosis and treatment planning. Further research and validation will aid in realizing the entire capability of the U-Net-GWBCO architecture, ultimately improving patient care and advancing medical imaging.

By achieving these advancements, the U-Net GWBCO model not only sets a new benchmark in medical image segmentation but also demonstrates its potential to transform clinical practice. The superior accuracy in delineating both pancreatic structures and tumors enhances diagnostic precision, leading to more tailored and effective treatment plans. This innovation holds the promise of significantly improving patient outcomes by enabling earlier and more accurate interventions. Additionally, the success of this model in integrating metaheuristic optimization techniques with U-Net underscores its scalability and applicability to other complex medical imaging tasks. As further research and validation continue to refine this approach, the U-Net GWBCO model is poised to become a cornerstone in the next generation of medical imaging technologies, driving forward both the science of image analysis and quality of patient care.

## Ethics statements

The present study did not involve human subjects, animal experiments, or data collected from social media platforms.

## CRediT authorship contribution statement

**Himali Ghorpade:** Conceptualization, Methodology, Data curation, Writing – original draft. **Shrikrishna Kolhar:** Conceptualization, Validation, Supervision. **Jayant Jagtap:** Writing – review & editing. **Jayasree Chakraborty:** Writing – review & editing.

## Declaration of competing interest

The authors declare that they have no known competing financial interests or personal relationships that could have appeared to influence the work reported in this paper.

## Data Availability

No data was used for the research described in the article.
